# Resilience among older adults with Type 2 Diabetes from the Look AHEAD trial

**DOI:** 10.1093/geroni/igab046.3278

**Published:** 2021-12-17

**Authors:** KayLoni Olson, Marjorie Howard, Jeanne Mccaffery, Gareth Dutton, Mark Espeland, Karen Johnson, Felicia Simpson, Rena Wing

**Affiliations:** 1 Alpert Warren Medical School of Brown University, Providence, Rhode Island, United States; 2 Wake Forest University Health Sciences, Winston Salem, North Carolina, United States; 3 University of Connecticut, Storrs, Connecticut, United States; 4 University of Alabama at Birmingham (UAB), Birmingham, Alabama, United States; 5 Wake Forest School of Medicine, Winston-Salem, North Carolina, United States; 6 University of Tennessee Health Science Center, Memphis, Tennessee, United States; 7 Department of Mathematics, Winston-Salem State University, Winston-Salem, North Carolina, United States; 8 The Miriam Hospital, Providence, Rhode Island, United States

## Abstract

There is growing interest in identifying factors protecting against aging-related decline. This cross-sectional study evaluated associations of self-reported resilience (ability to bounce back) with factors linked to aging-related decline among older adults with Type 2 diabetes (T2DM). Participants were 3,199 adults (72.2±6.2 years, 61% female, 61% white, BMI 34.2±8.2 kg/m2) enrolled in Look AHEAD (a multi-site RCT comparing weight loss to diabetes education among individuals with T2DM), who were followed observationally after the 10-year intervention was discontinued. The following items were assessed approximately 14.4yrs post-randomization: Brief Resilience Scale; overnight hospitalizations in past year; physical functioning measured objectively (gait speed, grip strength) and via self-report (Pepper Assessment Tool for Disability; Physical quality of life (QOL; SF-36)); a composite measure of phenotypic frailty based on having ≥3 of unintentional weight loss, low energy, slow gait, reduced grip strength, physical activity. Depressive symptoms (PHQ-9) and mental QOL (SF-36) were also measured. Logistic/linear regression was used to evaluate the association of these variables with resilience adjusted for age, race, and gender. Greater resilience was associated with lower BMI (p=.01), fewer hospitalizations (p=.02), better physical functioning (i.e., lower self-reported disability, better self-reported physical QOL, faster gait speed, greater grip strength and lower likelihood of meeting criteria for frailty; all p<.001), fewer depressive symptoms and greater mental QOL. Resilience is associated with better performance on indicators of overall functioning and risk for decline among older adults. Findings correspond with efforts to shift narrative on aging beyond ‘loss and decline’ to highlight opportunities to facilitate healthy aging.

